# *Bacillus cereus* for Controlling Bacterial Heart Rot in Pineapple var. MD2

**DOI:** 10.21315/tlsr2022.33.1.5

**Published:** 2022-03-31

**Authors:** Naimah Husin, Zaiton Sapak

**Affiliations:** 1 Faculty of Plantation and Agrotechnology, Universiti Teknologi MARA, Jasin Campus, 77300 Merlimau, Melaka, Malaysia; 2 Sustainable Crop Protection Research Group, Universiti Teknologi MARA, 40450 Shah Alam, Selangor, Malaysia

**Keywords:** *Bacillus cereus*, Bacterial Heart Rot, *Dickeya zeae*, Pineapple, *Bacillus cereus*, Reput Jantung Teras, *Dickeya zeae*, Nanas

## Abstract

Bacterial heart rot (BHR) disease caused by pathogenic bacteria, *Dickeya zeae*, is one of the destructive diseases of pineapple worldwide. This study explored the potential of *Bacillus cereus* against the BHR pathogen *in vitro* and *in vivo*. The BHR causal pathogen was isolated from symptomatic pineapple plants, demonstrating water-soaked and rotten basal tissues. Biological control agent (BCA) was isolated from asymptomatic pineapple leaves, later confirmed as *B. cereus*, and subsequently tested for the antagonistic activity against the BHR pathogen via disc diffusion assay and glasshouse trial. *B. cereus* showed the ability to inhibit the growth of BHR pathogen with 18.10 ± 0.36 mm of inhibition zone in diameter. The ability of *B. cereus* against the BHR pathogen was further confirmed via the glasshouse trial with five treatments. The results showed that treatments with *B. cereus* inoculation recorded lower disease severity index of 0.04 ± 0.01 than the positive control treatment with pathogen alone (0.53 ± 0.04). This finding indicated that *B. cereus* has a great potential as BCA against BHR disease in pineapple var. MD2, however, the effectiveness of this isolate needs to be further tested under actual field conditions.

## Abstract (fr)

Penyakit reput teras (BHR) yang disebabkan oleh bakteria patogen, *Dickeya zeae*, ialah salah satu penyakit yang merosakkan tanaman nanas di seluruh dunia. Kajian ini mengkaji potensi *Bacillus cereus* sebagai agen kawalan penyakit BHR secara *in vitro* dan *in vivo*. Patogen BHR dipencilkan daripada tumbuhan nanas yang mempunyai simptom penyakit seperti tisu daun menjadi lepuh, berair dan busuk. Ejen kawalan biologi diasingkan daripada daun nanas yang tidak mempunyai penyakit, dan dikenal pasti sebagai sebagai *B. cereus*, dan seterusnya diuji untuk aktiviti antagonis terhadap patogen BHR melalui melalui ujian *disc diffusion* dan penelitian di rumah kaca. *B. cereus* menunjukkan keupayaan untuk menghalang pertumbuhan patogen BHR dengan 18.10 ± 0.36 mm diameter zon perencatan. Keupayaan *B. cereus* terhadap patogen BHR telah disahkan lagi melalui penelitian di rumah kaca dengan lima rawatan. Keputusan menunjukkan rawatan dengan inokulasi *B. cereus* mencatatkan indeks keterukan penyakit yang lebih rendah sebanyak 0.04 ± 0.01 daripada rawatan kawalan positif dengan patogen sahaja (0.53 ± 0.04). Penemuan ini menunjukkan bahawa *B. cereus* mempunyai potensi yang besar sebagai BCA terhadap penyakit BHR dalam varian nanas MD2, bagaimanapun, keberkesanan pengasingan ini perlu diuji lagi di bawah keadaan lapangan sebenar.

**Highlight**

- *Bacillus cereus* has great potential as a biological control agent against *Dickeya zeae*, a causal pathogen of bacterial heart rot in pineapple.
- *Bacillus cereus* showed the ability to inhibit the growth of the bacterial pathogen in vitro.
- *Bacillus cereus* displayed great ability to reduce disease severity of bacterial heart rot in pineapple plants.

## INTRODUCTION

Pineapple (*Ananas comosus*) is one of the edible Bromeliaceae fruits commercially cultivated and produced in the tropic and subtropic regions. In 2017, in terms of global production volume, pineapple ranked second after mango with 27% of production volume (Altendorf 2019). The global pineapple production is projected by Food and Agriculture Organization (FAO 2020) to grow at 1.9% annually, reaching 31 million tons by 2028. Costa Rica and Brazil are the leading producers and exporters of pineapple. Pineapple is also widely cultivated in Southeast Asia including Malaysia, Thailand, the Philippines and Indonesia. Generally, commercial pineapple cultivars are grouped into four: Smooth Cayenne, Red Spanish, Queen and Abacaxi, with many varieties in each group (Thalip *et al*. 2015).

In Malaysia, there are 12 registered varieties of pineapple that are commercially grown either in small or large scales, including AC1-Moris, AC2-Sarawak, AC3-Gandul, AC4-Maspine, AC5-Josapine, AC6-Yankee, AC7-Moris Gajah, AC8-N36, AC9-MD2, AC10-View of Sunset and AC12-Keningau Diamond. A hybrid variety, MD2 has been acknowledged as a key crop and has become economically important for Malaysia. Among other varieties, MD2 is commercially grown in Johor, Perak, Selangor, Pahang, Pulau Pinang and Kedah to fulfill local and international market demands (Thalip *et al*. 2015). This pineapple variety possesses several good traits such as sweet taste, excellent sources of substantial calcium, potassium, glucose, the protein-digesting enzyme bromelain, fiber, vitamins A, B and C (Loh *et al*. 2017).

Like other crops, pineapple is also prone to many pests and diseases that cause huge yield losses. Many important diseases that have been reported associated with pineapple, and one of them is bacterial heart rot (BHR) caused a by bacterial pathogen previously identified as *Erwinia chrysanthemi*. Later, this pathogen was re-identified as *Dickeya zeae* based on a phylogenetic analysis using a multilocus sequence analysis by Ramachandran *et al*. (2015). In the pineapple farms, BHR disease could be noticed based on disease symptoms of watery rot. The initial disease symptom demonstrates a water-soaked lesion on the white basal portion of the central whorl (Joy & Sindhu 2012). Within 72 h, the light brown streaks form on the lamina and mesophyll, often filled with gas-forming blister-like lesions. The infection may then spread to the entire basal portion of all leaves of the central whorl before spreading to the whole length of the leaves, displaying an olive-green leaf colour and a bloated appearance (Joy & Sindhu 2012). As the infection progresses, a light brown exudate comes out of the blister, and leaves turn light brown to dark brown as the leaves slowly rot. Typically, the pineapple heart and stem can be easily detached from the lower portion of the plant around 1–2 weeks after initial symptoms. About 21 days after the initial infection, the whole infected area on the leaf eventually rots (Kaneshiro *et al*. 2008; Joy & Sindhu 2012).

Pineapple growers have been implemented several practices to control the disease, such as selecting disease-free planting materials, developing a good drainage system to avoid waterlogged, planting beds and applying chemical pesticides (Malaysian Pineapple Industry Board, MPIB 2020). Growers use chemical pesticides such as malathion to treat the planting materials before planting and benomyl after the disease symptoms appear in the field (MPIB 2020; Sidik & Sapak 2021). The other chemical pesticides are commonly used by pineapple growers, such as copper hydroxide, mancozeb, and thiram (Thalip *et al*. 2015). However, the intensive uses of these chemical pesticides could lead to several issues such as pesticide resistance (Hawkins *et al*. 2019), environmental contamination with pesticide residues in soil and food (Navarro *et al*. 2021; Syafrudin *et al*. 2021), and human health concerns (Ali *et al*. 2021). Therefore, an alternative control method that can reduce the heavy reliance on chemical pesticides is crucially needed to control this disease in pineapple farms. A biological control method is one of the alternative control strategies to be explored for controlling BHR. Beneficial microbes isolated from healthy plants could offer huge potential as Biological control agents (BCAs) to control the disease. Most BCAs can suppress the pathogen via their antagonistic mechanisms (Kaneshiro *et al*. 2008). To the author’s knowledge, in Malaysia, the control of BHR in the pineapple with BCAs is not yet fully explored and established. Therefore, this study aim was to evaluate the potential of *Bacillus cereus* isolated from asymptomatic pineapple leaves as a BCA for controlling BHR disease in pineapple var. MD2 as the disease was found to be more severe in this variety.

## MATERIALS AND METHODS

### Diseased and Healthy Plant Samples

Twenty samples of diseased pineapple plants var. MD2 with BHR symptoms were randomly collected at Kluang, Johor, in September 2014. These diseased plant samples were brought to the Plant Pathology Laboratory at Puncak Alam Campus, UiTM, for isolation and confirmation of the causal pathogen of the disease. Meanwhile, 20 healthy pineapple plants from the same variety were also collected from the same place to isolate BCAs. Every single plant sample was kept in different plastic bags and placed in a cooler box to maintain the freshness of the leaves during the transportation to the laboratory.

### Isolation of the Causal Pathogen and Biological Control Agents

Isolations of BHR pathogen and BCA were conducted based on the method of Ku Asmah and Sapak (2020). The infected and healthy pineapple leaves were cut into small segments of 1 cm in length. Then, the leaf segments were surface sterilised with 1% sodium hypochlorite for 2 min, followed by 95% ethanol for 1 min, and then rinsed in sterile distilled water twice before placed them on sterile filter papers to dry excessive water from the sterilisation process. The leaf segments were recut into small sections (0.5 cm × 0.5 cm) with a sterilised scalpel before placing them onto nutrient agar (NA) in the Petri plates. The plates were properly sealed with parafilm and incubated for 48 h at 28 ± 2°C. The bacterial pathogen that grown out from the infected leaf tissues was isolated and purified into a pure culture. The procedure similar to the potential BCAs grown from the healthy leaf tissues were isolated and purified into pure cultures for further evaluation. The bacterial isolate from the infected plant samples was confirmed as a main causal pathogen of BHR via Koch’s Postulates and Biolog^®^ GEN III Microplate Identification System.

### Screening and Identification of Biological Control Agent *in vitro*

Twenty-five bacterial isolates from the healthy leaf segments were screened for their potential as BCA against the BHR pathogen using an antibiotic disc diffusion assay (“Kirby-Bauer” assay) (Balouiri *et al*. 2016). The assay was conducted by evenly streaking the bacterial pathogen on NA in the Petri plates and incubated for 24 h at 28 ± 2°C. The beneficial bacterial isolates were then taken out as discs from the fully grown culture of 48 h-old using a sterile cork borer (0.2 cm) and placed the discs onto the pathogen plates. The plates were further incubated at 37 ± 2°C for 24 h. After the incubation period, the diameter of the inhibition zone produced surrounding the discs was measured. The biggest diameter of the inhibition zone would indicate the strongest BCA to suppress the pathogen growth. Bacterial isolate with the biggest inhibition zone was selected and subjected to further tests as potential BCA. Identification of the potential BCA was performed through morphological characteristics including Gram stain, cell shape, single colony colour, form, elevation and margin edge and confirmed by Biolog^®^ GEN III Microplate Identification System (Techno Science, Malaysia).

### Evaluation of *Bacillus cereus* against BHR Pathogen *in vivo*

Pineapple var. MD2 suckers were used in this *in vivo* study. Fifty pineapple suckers certified disease-free and an approximate weight of 1.5–2.0 kg each were purchased from KOSAS Sdn. Bhd. and grown in polybags (35 cm × 51 cm) for three months in the glasshouse at the Faculty of Plantation and Agrotechnology, UiTM, Puncak Alam, Selangor. They were watered and fertilised according to the Malaysian Pineapple Industry Board (MPIB). All the pineapple plants were monitored daily to ensure no diseases before inoculation with BHR pathogen and selected BCA. In this experiment, the potential of BCA to inhibit the growth of pathogen and disease symptoms was studied with five designated treatments (Table 1). In the first treatment (T1), pineapple plants were inoculated with the BHR pathogen suspension of 10^8^ CFU mL^−1^ and BCA suspension of 10^8^ CFU mL^−1^ on the same day. The pineapple plants in the second treatment (T2) were established with BCA suspension of 10^8^ CFU mL^−1^ for one week and then followed by the BHR pathogen suspension (10^8^ CFU mL^−1^). Both pathogen and BCA were inoculated at the centre of the pineapple plants to accelerate the infection process and the effectiveness of BCA. The treatments of T3, T4 and T5 served as positive (pathogen alone), negative (BCA alone), and healthy plant (without pathogen and BCA) controls, respectively. Each treatment consisted of 10 plants, and the plants were arranged in a completely randomised design (CRD) in the glasshouse with uniform conditions. Disease symptoms development on the inoculated pineapple plants were observed weekly for up to six weeks and rated based on the disease scores of 0 to 4 (Table 2). The scale used was modified from the scale for soft rot disease by Lee *et al*. (2006) based on the area of rotten tissues. In addition, the disease severity index (DSI) was then calculated using a formula of Sapak *et al*. (2008) as follows:


$$\text{DSI}=\frac{\text{sum of all scores}}{\text{total scores}}\times \text{maximum disease score}$$

All the collected data were analysed by ANOVA with means compared by the LSD (*p* ≤ 0.05).

### Colonisation of *B. cereus* in Pineapple Leaf Tissues

At the final stage of the glasshouse experiment, five pineapple plants treated with *B. cereus* (BC3) from T1, T2 and T4 were randomly collected to assess the colonisation of BC3 in the pineapple leaf tissues. Four leaf samples per plant were cut and washed under running tap water to remove any debris. Then, the leaves were cut into small segments and surface sterilised by 1% sodium hypochlorite solution for 10 min and 70% ethanol for 1 min, followed by rinsing with sterile water twice. The leaf segments were crushed using a sterilised mortar and pestle, then 1 g of the crushed leaves were added into 20 mL of saline water in universal bottles and kept for 1 h. A serial dilution was performed for each sample and 0.1 mL of the solution was then directly plated onto NA for bacteria colony counting (Mattos *et al*. 2008). The colony growth on the NA plate was counted to examine the colonisation of BC3 in the pineapple leaf tissues for each treatment.

## RESULTS AND DISCUSSION

### Isolation and Identification of the Causal Pathogen

**Figure 1:**
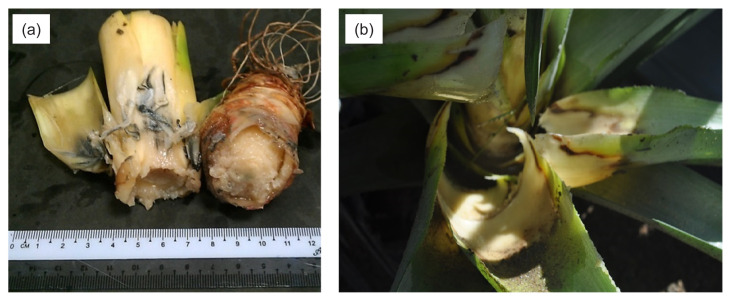
The infected pineapple plant displayed BHR symptoms of (a) water-soaked, rotten tissue, bad odour and (b) the leaves are easily detached from the plant.

Eleven bacterial isolates were isolated from the diseased pineapple leaves. Out of 11 bacterial isolates, five were Gram-negative and cocci-shaped, and two were Gram-negative and rod-shaped. Meanwhile, two isolates were Gram-positive and rod-shaped, and the remaining two isolates were Gram-positive and cocci-shaped. Aeny *et al.* (2020) described BHR pathogen, *D. zeae* colonies on media as circular, convex, cream white milk-coloured, with diameter colonies ranging from 1 mm to 2 mm. In our observation, the colonies of isolate coded as B1 on NA appeared as described. Meanwhile, microscopic features of *D. zeae* can be characterised as Gram-negative, motile rod-shaped, non-sporing and occurring singly or in pairs. The isolate B1 with the morphological characteristics of *D. zeae* was further confirmed with the pathogenicity test result. The infected pineapple plants displayed typical symptoms of BHR, which were water-soaked lesions, foul odour, and the infected leaves were easily detached from the plant central (Fig. 1). The isolate was then confirmed as *D. zeae* by Biolog^®^ GEN III Microplate Identification System.

### Isolation, Screening and Identification of Biological Control Agent

Bacterial isolation from healthy pineapple leaves discovered 25 isolates, with 10 of them were Gram-positive and 15 isolates were Gram-negative. Eighteen bacterial isolates were cocci-shaped, and seven isolates were observed as rod-shaped. The highest potential BCA was based on the results of the bacterial activity assay which showed that the most potential BCA was obtained from isolate coded as BC3 with the diameter of inhibition zone was 18.10 ± 0.36 mm. The other bacterial isolates showed less effectiveness with a diameter of inhibition zone between 5 mm to 8 mm (Table 3). The colonies of BC3 isolate grown on NA can be characterised as white, irregular, flat and undulated colonies and the microscopic features can be described as Gram-positive, rod, motile, facultative, aerobic and forming spore. These characterisations were similar, as described by Stabb *et al*. (1994). The isolate BC3 was confirmed as *B. cereus* by the Biolog^®^ identification result.

### The Effectiveness of *B. cereus* to Control BHR Disease in the Glasshouse

The initial disease symptoms of BHR appeared after a week of inoculation, and the DSI was observed in the T3 (pathogen alone) with the highest DSI of 0.53 ± 0.04 compared to T1 (0.05 ± 0.01) and T2 (0.04 ± 0.01) (Table 4). The DSI values increased with time in all treatments; however, at the slow progress BC3. As expected, after six weeks of observation, the highest DSI was recorded in T3. Meanwhile, the lowest DSI was obtained from T2 with a DSI of 0.17 ± 0.03. Nevertheless, there were no significant differences in DSI between T2 and T1 (Table 4). These findings suggested that BC3 could suppress the pathogen and delay the disease development progress regardless of BC3 application time either before or after the pathogen. The potentials of *B. cereus* as BCA have been reported by many studies, such as for controlling bacterial leaf blight (Ahmed *et al*. 2020) and blast (Huang *et al*. 2020) in rice, tomato wilt disease and root knot nematodes (Wang *et al*. 2019), leaf blight of lily (Huang *et al*. 2005), early leaf-spot of peanuts (Kokalis-Burelle *et al*. 1992), damping-off disease in alfalfa seedlings (Handelsman *et al*. 1990), and many other crop diseases. To the authors’ knowledge, there is a limited number of published reports on *B. cereus* as a biological control for pineapple diseases, except by Leite *et al*. (2016) used antimicrobial of *B. cereus* that was isolated from pineapple pulp for controlling food spoilage bacteria in pineapple pulp. Our finding revealed that *B. cereus* isolated from asymptomatic pineapple plants has a great potential as a BCA for controlling BHR as the disease severity can be limited by the BC3. Some studies found that *B. cereus* is able to produce antimicrobial substances to successfully kill plant pathogens such as cyclodextrin glycosyltransferase (Zhou *et al*. 2021), kanosomine (Milner *et al*. 1996), Zwittermicin A and Antibiotic B (aminoglycoside) (Silo-Suh *et al*. 1998), and bacteriocin-like inhibitory substance (Risøen *et al*. 2004).

### Colonisation and Recovery of *B. cereus* in Pineapple Leaf Tissues

The colonisation and establishment of *B. cereus* (BC3) in the pineapple leaves were quantified based on colony-forming units per g tissue (CFU g^−1^). The results showed the highest recovery of BC3 in the T4 with 6.6 × 10^7^ CFU g^−1^ followed by T1 (5 × 10^7^ CFU g^−1^) and T2 (4.2 × 10^7^ CFU g^−1^). These findings suggested that the recovery of BC3 at a high number of colonies in the treatments could be the main reason for reducing BHR disease. Many studies have been reported the capability of *B. cereus* to colonise and survive in plant tissues. Ku *et al*. (2018) mentioned that *B. cereus* was able to colonise soybean roots after three days of application and Wang *et al*. (2019) revealed that the application of *B. cereus* in tomato plants was able to colonise and inhibit the growth of bacterial wilt pathogen, *Ralstonia solanacearum*. A recent study by Arif *et al*. (2021) found that *B. cereus* applied in *Brassica campestris* could colonise and inhibit the growth of clubroot pathogen. Yin *et al*. (2021) reported that *B. cereus* applied in cucumber plants was able to colonise against *Meloidogyne incognita* well.[Fig f2-tlsr-33-1-77]

## CONCLUSION

The findings of this study revealed that *B. cereus* (BC3), which was isolated from asymptomatic pineapple leaves var. MD2 was able to inhibit the growth of BHR pathogen and could be a promising BCA. However, further investigations are still required, including rigorous field assessment trials to confirm the effectiveness of this BCA under real conditions.

## Figures and Tables

**Figure 2 f2-tlsr-33-1-77:**
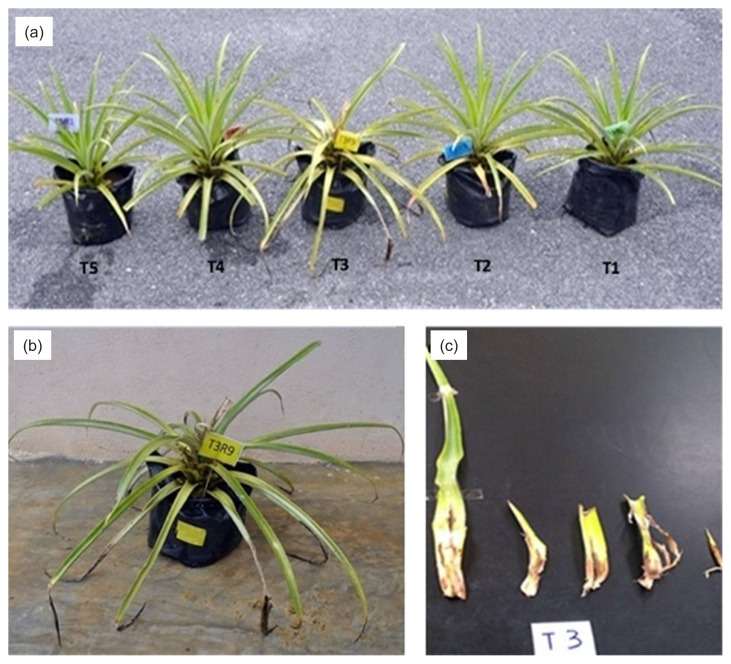
(a) Infected pineapple plant with pathogen alone (T3) showed severe symptoms of BHR compared to other treatments as the leaves collapsed (b) and easily to be detached from the central plant (c).

**Table 1:** Five treatments used to evaluate the effectiveness of *B. cereus* (BC3) against bacterial heart rot pathogen in pineapple var. MD2 in the glasshouse trial.

Treatment	Description
T1	Pathogen and BC3 (Apply at the same day)
T2	BC3 (Apply a week before) and pathogen
T3	Pathogen only (Positive control)
T4	BC3 only (Negative control)
T5	Healthy Plant Control (without pathogen and BCA)

**Table 2:** A modified disease scoring based on Lee *et al*. (2006) used to measure the disease severity of infected pineapple plants with bacterial heart rot symptoms.

Score	Symptoms
0	No visible symptoms with 0% area infected
1	0.5 cm–1.0 cm of the segment (starting from the inoculated position) rotted with 25%
2	1.0 cm–2.5 cm of the segment rotted 50%
3	2.5 cm–4.0 cm of the segment rotted 75%
4	More than 4 cm or the whole segment rotted 100%

**Table 3:** Twenty-five bacterial isolates from healthy pineapple leaves with different characteristics and inhibition zone diameter.

Code	Gram stain, cell shape, colony colour, form, elevation and margin edge of single colonies)	Inhibition zone of bacteria (mean of 3 replicates ± SD mm)
BC1	Positive, cocci, white, circular, flat and entire	6.33 ± 0.58
BC2	Negative, cocci, white, irregular, flat and undulated	7.33 ± 0.58
BC3	Positive, rod, white, irregular, flat and undulated	18.10 ± 0.36
BC4	Positive, rod, cream, irregular, flat and undulated	4.83 ± 0.76
BC5	Positive, rod, cream, circular, flat and undulated	5.00 ± 0.20
BC6	Negative, cocci, white, circular, raised and entire	5.20 ± 0.20
BC7	Negative, cocci, white, circular, flat and entire	5.20 ± 0.26
BC8	Negative, cocci, white, circular, convex and entire	5.50 ± 0.50
BC9	Positive, cocci, white, circular, flat and entire	4.77 ± 0.25
BC10	Positive, cocci, cream, irregular, flat and undulated	5.17 ± 0.21
BC11	Positive, cocci, white, irregular, raised and undulated	5.77 ± 0.25
BC12	Negative, cocci, white, irregular, flat and entire	4.77 ± 0.25
BC13	Negative, cocci, white, circular, flat and entire	5.10 ± 0.10
BC14	Positive, rod, white, irregular, raised and lobate	5.10 ± 0.26
BC15	Negative, cocci, white, circular, flat and undulated	4.93 ± 0.21
BC16	Negative, cocci, white, circular, flat and entire	7.13 ± 0.32
BC17	Negative, cocci, white, irregular, flat and lobate	5.20 ± 0.20
BC18	Positive, cocci, cream, irregular, flat and undulated	9.03 ± 0.15
BC19	Negative, rod, white, irregular, flat and undulated	6.03 ± 0.15
BC20	Negative, rod, white, circular, raised and entire	5.03 ± 0.14
BC21	Negative, cocci, white, circular, flat and entire	4.97 ± 0.06
BC22	Positive, cocci, white, circular, flat and entire	4.93 ± 0.12
BC23	Negative, cocci, cream, irregular, raised and undulated	5.90 ± 0.36
BC24	Negative, cocci, white, circular, flat and entire	7.03 ± 0.15
BC25	Positive, cocci, white, circular, convex and entire	8.03 ± 0.15

**Table 4:** Disease severity index recorded in three treatments of pineapple plants inoculated with bacterial heart rot pathogen and *B. cereus* for six weeks.

Observation weeks	Disease severity index		

	T1 *Mean ± SE	T2 *Mean ± SE	T3 *Mean ± SE
1	0.05 ± 0.01^b^	0.04 ± 0.01^b^	0.53 ± 0.04^a^
2	0.09 ± 0.02^b^	0.06 ± 0.02^b^	0.64 ± 0.03^a^
3	0.10 ± 0.03^b^	0.10 ± 0.02^b^	0.73 ± 0.04^a^
4	0.12 ± 0.03^b^	0.10 ± 0.02^b^	0.76 ± 0.03^a^
5	0.16 ±.0.03^b^	0.13 ± 0.03^b^	0.79 ± 0.03^a^
6	0.21 ± 0.03^b^	0.17 ± 0.03b	1.00 ± 0.01^a^

*Notes*:

* Mean ± Standard Error (SE) between treatments (column) indicated with the different superscript letter are significantly difference at *p* ≤ 0.05.
